# Premenstrual Syndrome: Existence, Knowledge, and Attitude Among Female University Students in Karachi

**DOI:** 10.7759/cureus.2290

**Published:** 2018-03-08

**Authors:** Aleena Mohib, Amara Zafar, Areeba Najam, Hafsa Tanveer, Rehana Rehman

**Affiliations:** 1 Dow Medical College, Civil Hospital Karachi, Dow University of Health Sciences, Karachi, Pakistan; 2 Department of Biological and Biomedical Sciences, Aga Khan University, Karachi.

**Keywords:** premenstrual syndrome, prevalence, knowledge, attitude, women's health

## Abstract

Objective

The aim of this study was to investigate the existence, knowledge, and the attitude of female students towards premenstrual syndrome (PMS).

Methods

This cross-sectional study was conducted in three universities in Karachi, Pakistan. A total of 448 female students participated in the study. The clinical criterion of American College of Obstetricians and Gynecologists (ACOG) for PMS was used to assess the prevalence of PMS in the participants. The questionnaire was set in four parts, one each to assess the knowledge, the attitude, and practices regarding PMS and one to assess the gap between self-perceived PMS and actual PMS. Data were analyzed descriptively using the Statistical Package for the Social Sciences (SPSS), version 20 (IBM SPSS Statistics, Armonk, NY).

Results

The majority (96.4%) of female students were aware of PMS, while only 19% females knew about premenstrual dysphoric disorder. The self-reported prevalence of PMS was 79.5% and the prevalence using the ACOG criteria was 23.9%. Common symptoms were irritability, angry outbursts, depression, breast tenderness, and gastrointestinal problems. More than half (60.4%) of the participants reported PMS disturbed their normal routine, while 81.5% reported stress exacerbated their symptoms. The majority (77.5%) of women believed PMS was a significant issue to be discussed but 49.4% did not take treatment for their PMS.

Conclusion

There is a significant impact of PMS in the lives of Pakistani women, and it is a common problem all over the globe. Despite the growing awareness, there remains a considerable deficiency of knowledge about the necessity to consult a doctor or seek treatment for their symptoms.

## Introduction

The majority of women of reproductive age experience physical or emotional symptoms before the onset of menstruation [1]. Amongst those, some women are so severely affected that it interferes with their mental health, interpersonal relationships, and studies [1]. It has also been found that the prevalence of premenstrual syndrome (PMS) is higher in unmarried women, in women aged 35-44 years, and in women who belong to a low socioeconomic group living in socially deprived areas [2-3].

Several studies have reported multiple risk factors associated with PMS, where stress, age, body mass index, and marital status were found to exacerbate the symptoms [4]. A study also found a direct association of PMS with parity, which was low in the low parity group [3]. Premenstrual symptom frequency was also significantly associated with maternal history of PMS [5]. While all these factors show significant correlation with PMS, caffeine intake was reportedly not associated with it [6]. 

The study conducted by Pal et al. on Pakistani women states that physical symptoms predominate in the premenstrual experience of the Pakistani women and described abdominal bloating and cramps, irritability, and mood swings as the more common symptoms experienced by the women [7]. Another study reported joint pain, muscle pain, back pain, and breast tenderness as the most prevalent symptoms [8]. Several authors found anxiety, depression, fatigue, and anger as the most frequently reported symptoms [9]. Studies also suggested skin disorders, swelling of extremities, gastrointestinal problems (like decreased appetite), and headaches as symptoms experienced by women before menstruation [10-11]. In order to alleviate these symptoms, some of the women use some self-treatment strategies, amongst which the most frequently used are taking analgesics, increasing hot fluid intake, wearing warm clothes, and lying down on the abdomen, while majority refer not to seek any treatment for their complaints [3-4].

Another clinical entity called premenstrual dysphoric disorder is a less common but a far more serious condition than PMS. This disorder also consists of affective and behavioral symptoms during the late luteal phase of the ovulatory cycle. Even then, only a few women are reported to experience premenstrual dysphoric disorder [12].

No study has been previously conducted in Pakistan that compares the percentage of women who are experiencing PMS with those who perceive they are experiencing the syndrome, although they do not meet the clinical criteria of PMS. Along with this, the purpose of this study is to see how many females going to university are aware of the risk factors and the treatment options. The aim is to find out their perception of the situation and whether PMS has a significant or insignificant impact on the lives of women going to a university.

## Materials and methods

This cross-sectional study was conducted on female university students in Karachi from September 1, 2017 to December 22, 2017. Participants were selected by random sampling between the ages of 18 to 30 years from different universities (namely, Dow University of Health Sciences (DUHS), Jinnah Sindh Medical University (JSMU), and Institute of Business Administration (IBA)) with the majority of the sample being medical students due to majority of the data collectors based in medical universities. The sample size of 448 was calculated using open EPI Info™ software (http://www.cdc.gov/epiinfo/index.html) sample size calculator with 95% confidence interval and 5% margin of error. Women with menstrual irregularities in the past six months and known cases of any psychological or medical disorder were excluded with menstrual irregularities being defined as cycles less than 26 days or longer than 35 days. 

A structured questionnaire was designed after thorough literature searches, questions were adapted and modified from previously published studies as per the requirement, and questions were added which were considered relevant. The questionnaire was thoroughly reviewed by two proficient doctors and a pilot tested on 10 participants for relevance, coherence, and clarity before being administered to the study. Data were collected using the self-administered anonymous questionnaire after obtaining informed consent.

The questionnaire was comprised of six parts. The first part included questions on demographic data, e.g., age, marital status, education, income, smoking, and exercise habits. The second part consisted of a series of questions inquiring if the women experienced PMS, their symptoms and onset, and duration of those symptoms. Women were also asked regarding the impact PMS had on their lives, including the effect of stress on the severity of PMS. The third part included questions about their knowledge about PMS, including its risk factors and treatment options. Furthermore, we questioned the women on their knowledge about the premenstrual dysphoric disorder. In the fourth part of the questionnaire, the participants were questioned about their practices to relieve their symptoms of PMS. In the next section, participants were questioned regarding their attitude towards PMS, their reservations, and opinions regarding a discussion about PMS. In the final section, the participants were asked to record their premenstrual symptoms for the next two cycles.

To diagnose PMS, the criteria of American College of Obstetricians and Gynecologists (ACOG) for PMS [13] was used which included at least one of the affective and somatic symptoms that must be present in the five days before menses for at least three previous menstrual cycles in a row, end within four days after of the onset without recurrence until at least day 13 of the cycle, and must be present in the absence of any pharmacologic therapy, hormone ingestion, or drug or alcohol use. The symptoms must occur reproducibly during two cycles of prospective recording. The patient must exhibit identifiable dysfunction in social, academic, or work performance.

Data were analyzed descriptively using IBM Statistical Package for the Social Sciences (SPSS) version 20.0 (IBM SPSS Statistics for Windows, Armonk, NY) and tables were constructed using Microsoft Excel® 2016 (Microsoft Corp., Redmond, WA). Results for a quantitative variable were presented as frequencies and percentages.

## Results

The mean age of the participants was 21.52 ± 1.69 years. Table 1 shows that the majority (94.9%) of the females in our study were single, 80.8% were enrolled in medical universities, 95.3% were pursuing a bachelor's degree, and 85.0% of the female’s monthly household income was more than 50,000 rupees (PKR) ($452 USD). The majority (96.4%, n = 432) of females had heard of PMS. Almost three quarters (72.8%, n = 326) females correctly defined PMS. More than one-third (37.9%, n=170) females said that they knew the risk factors of PMS. About two-fifths (19%, n = 85) of the females knew about premenstrual dysphoric disorder.

**Table 1:** Socio-demographics 50,000 Pakistani rupees (PKR) = $452 United States dollars (USD) N: number

Characteristics	N (%)
Monthly Income	
Family income > 50,000 PKR/month	381 (85.0%)
Family income < 50,000 PKR/month	67 (15.0%)
Education Level	
Bachelors	427 (95.3%)
Post-graduation/Masters	21 (4.7%)
University Field	
Medical	362 (80.8%)
Non-medical	86 (19.2%)
Marital Status	
Single	425 (94.9%)
Married	22 (4.9%)
Divorced	1 (0.2%)

**Figure 1:**
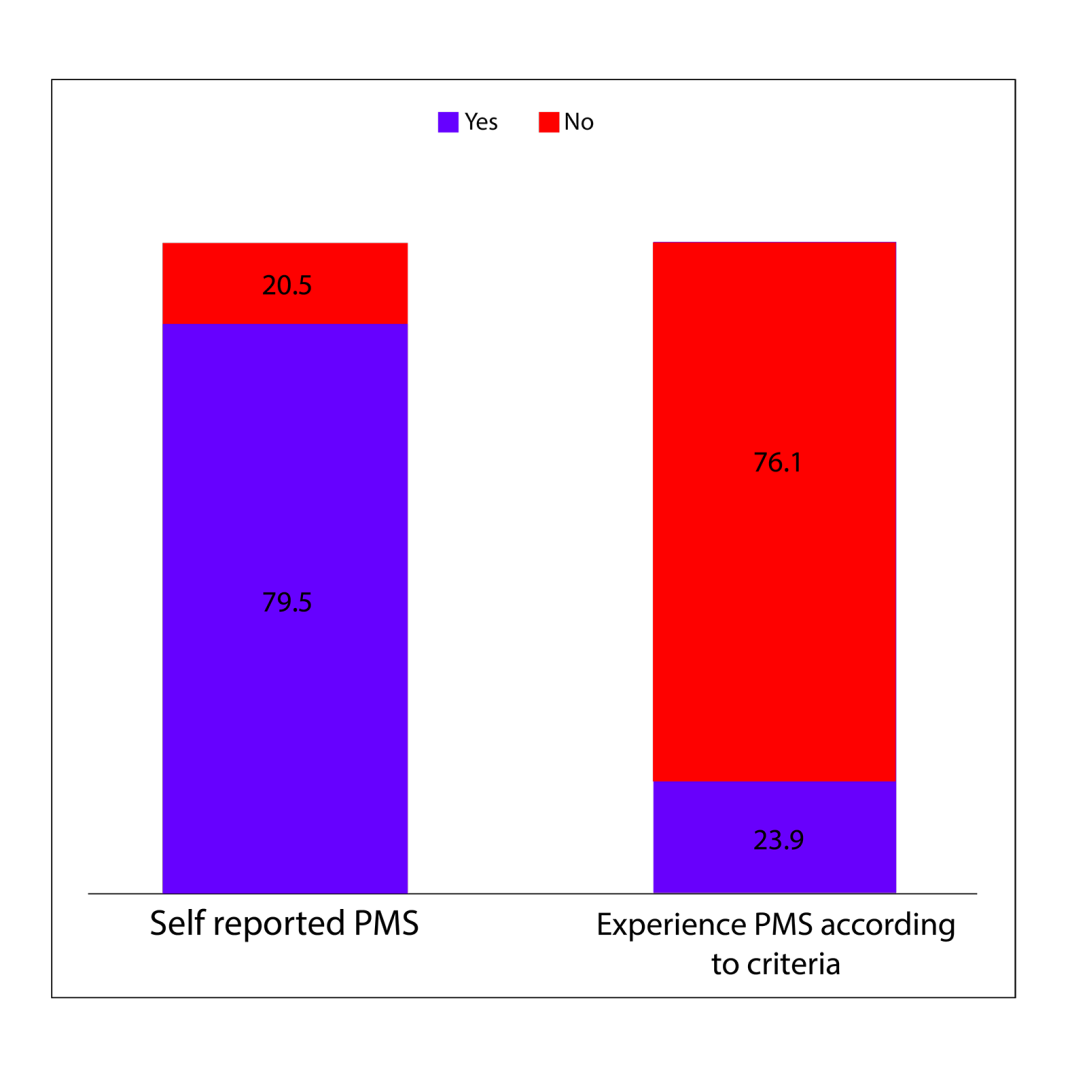
Comparison Between Self-reported Premenstrual Syndrome (PMS) and Premenstrual Syndrome (PMS) According to the American College of Obstetrics and Gynecology (ACOG) Criteria

Figure 1 shows that 356 females (79.5%), when asked, said that they experience PMS, while 92 (20.5%) said they do not experience PMS. When evaluated according to the criteria, it turned out that only 107 females (23.9%) actually experienced PMS. A profound gap was found between self-perceived PMS and actual PMS.

Table 2 shows the symptoms of PMS among the 356 females who self-reported to have PMS. Irritability was the most common complaint with 81.7% females reporting it as one of their symptoms. Angry outbursts (66.9%), depression (53.1%), anxiety (46.9%), skin problems (42.7%), breast tenderness (39.6%), gastrointestinal problems (39.3%), social withdrawal (37.1%), abdominal swelling (33.7%), changes in sleep (33.4%), headache (21.9%), and swelling of arms and legs (8.1%) were other reported complains.

**Table 2:** Symptoms of Premenstrual Syndrome (PMS)

Symptoms of PMS	N (%)
Irritability	291 (81.7%)
Angry outbursts	238 (66.9%)
Depression	189 (53.1%)
Anxiety	167 (46.9%)
Skin problems, like acne	152 (42.7%)
Breast tenderness	141 (39.6%)
Gastrointestinal problems	140 (39.3%)
Social withdrawal	132 (37.1%)
Abdominal swelling	120 (33.7%)
Changes in sleep	119 (33.4%)
Headache	78 (21.9%)
Swelling of arms and legs	29 (8.1%)

Table 3 describes how PMS affects the normal life of females who experience PMS. More than half (60.4%) said PMS affected their normal routine. About one-third of the females reported missing school, work, or a social event due to their PMS. The majority (81.5%) of females reported that their PMS was exacerbated by stress. 

**Table 3:** Premenstrual Syndrome (PMS) and Normal Life N: number

How PMS affects normal life	N (%)
PMS disturbs normal routine	215 (60.4%)
Missed school or work due to PMS	126 (35.4%)
Missed social event due to PMS	143 (40.2%)
Stress exacerbates PMS	290 (81.5%)

Table 4 describes the attitude of females in our sample towards PMS. About two-thirds of the females believed that PMS/menstrual leave should be an option at the university and workplace. More than two-thirds of the females said that they are likely to talk to their family/spouse about PMS. When asked, only about a third of the females said that they are likely to consult a doctor for more information and a checkup for PMS. Three-quarters of the female sample said PMS was significant enough of an issue to be discussed.

**Table 4:** Attitude Towards Premenstrual Syndrome (PMS)

Attitude towards PMS	N (%)
Should PMS/menstrual leave be an option at university?	276 (61.6%)
Should PMS/menstrual leave be an option at the workplace?	297 (66.3%)
Are you likely to talk to your family/spouse about PMS?	309 (69.0%)
Are you likely to consult a doctor for a check-up for PMS?	166 (37.1%)
Do you think PMS is significant an issue to be discussed?	347 (77.5%)

Table 5 lists the options used by the women experiencing PMS in order to relieve their symptoms. Half of the women said they did nothing to relieve their PMS symptoms. Using analgesics was the leading treatment opted by the females in our sample for relieving their PMS symptoms while doing exercise was the second most common remedy used with one-fifth of the women opting it. Traditional remedies were being used by 13.8% of the females. Use of vitamin supplements (5.1%), homeopathic medications (1.4%), and anti-depressants (0.3%) were less common among the females in our sample.

**Table 5:** Treatments Options in Use for Premenstrual Syndrome (PMS) N: number

For relieving of symptoms of PMS	N (%)
Painkillers	146 (41.0%)
Homeopathic medication	5 (1.4%)
Vitamin supplements	18 (5.1%)
Traditional remedies	49 (13.8%)
Exercise	63 (17.7%)
Antidepressants	1 (0.3%)
Do nothing	176 (49.4%)

## Discussion

A significant fraction of women in Pakistan are afflicted by PMS [7]. In our society, menstruation and related subjects are considered taboo and women are made to believe that PMS is not a significant enough issue to be discussed or to seek help for, even if it is having a negative impact on their lives. The majority of the females in our study were aware of PMS and fewer knew about its risk factors, as compared to a study done in three metropolises of Pakistan where 98.8% of women were unaware of the phenomenon of PMS [7]. This substantial difference could be explained by the fact that the participants of our study were mostly medical students and thus were familiar with PMS. However, despite their medical background, only 19% of women knew about premenstrual dysphoric disorder. This goes on to show that there is still a deficiency of knowledge regarding common disorders afflicting women’s health.

There is no single tool to evaluate the prevalence of PMS, owing to the differences in diagnostic criteria, sampling, and data collection methods between studies conducted on PMS. This gives rise to discrepancies in results, making an adequate comparison of the prevalence of PMS difficult and becoming a limitation. Our study sought to differentiate between the number of women who believed that they experienced PMS and the women who actually experienced PMS according to the diagnostic criteria set by the ACOG for PMS. In our study, the self-reported prevalence of PMS was almost thrice the rate as diagnosed by means of the ACOG criteria, which is a staggering difference. A study in Brazil reported a similar trend with the prevalence of self-reported PMS being much higher than the prevalence of PMS determined using the modified Diagnostic and Statistical Manual of Mental Disorders, 4th Edition (DSM-IV) criteria for PMS [11]. This finding is crucial in achieving an understanding regarding the awareness and perception of PMS amongst women. 

PMS is usually described as a constellation of both somatic and affective symptoms manifesting prior to the occurrence of menstruation and resolving with the onset of menstruation or within few days of menstruation [11]. The study in hand found affective symptoms to be more common than physical symptoms, like irritability, angry outbursts, and feelings of depression, while skin problems and mastalgia were the most common physical symptoms. Our findings agree with studies done in Malaysia [14] and Thailand [15], whereas a study conducted in Iran reported tiredness and depressed mood being the more prominent symptoms [16].

It is no surprise that PMS causes significant effects not only on women’s normal daily routines but also their occupational and social life [17]. The results of our study are in accordance with those reported in earlier studies, which also recognized PMS as a significant and potentially disabling condition [18-19]. Furthermore, with stress being an inadvertent aspect of university life, it is necessary to assess its relationship with PMS. A study in Iran studied the relationship between work-stress and PMS and reported a positive association, similar to the results of our study in which 81.5% of participants reported that stress exacerbated their PMS [20].

In a study conducted in Karachi, the largest city of Pakistan, many participants reported being fearful at their first experience of menstruation, while more than half did not take baths during menstruation [21]. However, a positive attitude towards PMS was noted in our study with 77.5% of women believing that PMS is a significant issue to be discussed and many said that an option to take leave from work or school due to PMS should be available to women. Despite this encouraging response, almost half of the women reported they take no treatment to relieve their symptoms. This result is comparable to the findings of a national survey done in Spain where only 18.7% of women sought medical advice for PMS [22]. The findings could be explained by the fact that the condemnation of the subject of menstruation in our society has created an equally negative attitude in young females towards menstruation and related matters; this has created a hesitancy in consulting a doctor or seeking treatment for it, as they do not consider PMS a serious issue but just a normal part of their lives.

One of the limitations of this study is the use of the self-reported method of data collection. This can result in an overestimation of the prevalence of PMS. Another limitation is the small and selective sample size which was not evenly distributed. Hence, we were not able to compare the prevalence of PMS and differences between different socio-economic background and their marital status.

## Conclusions

This research concludes that PMS is a common problem in our part of the world affecting the quality of life of women significantly. Despite the growing awareness, there remains a considerable deficiency of knowledge about the necessity to consult a doctor or seek any treatment for their symptoms. Further research is required on a larger population and including women from various socio-economic backgrounds to better assess the situation and strategize to manage this rising problem. The majority of females said that stress exacerbates their PMS, and stress is a prevalent condition in our society. It is important that a healthy culture is promoted which is stress-free in order to avoid the symptoms of PMS, which tend to disturb normal routines and reduce productivity.
